# A rough set-based measurement model study on high-speed railway safety operation

**DOI:** 10.1371/journal.pone.0197918

**Published:** 2018-06-07

**Authors:** Qizhou Hu, Minjia Tan, Huapu Lu, Yun Zhu

**Affiliations:** 1 School of Automation, Nanjing University of Science and Technology, Nanjing, Jiangsu, China; 2 Institute of Transportation Engineering, Tsinghua University, Beijing, China; Southwest University, CHINA

## Abstract

Aiming to solve the safety problems of high-speed railway operation and management, one new method is urgently needed to construct on the basis of the rough set theory and the uncertainty measurement theory. The method should carefully consider every factor of high-speed railway operation that realizes the measurement indexes of its safety operation. After analyzing the factors that influence high-speed railway safety operation in detail, a rough measurement model is finally constructed to describe the operation process. Based on the above considerations, this paper redistricts the safety influence factors of high-speed railway operation as 16 measurement indexes which include staff index, vehicle index, equipment index and environment. And the paper also provides another reasonable and effective theoretical method to solve the safety problems of multiple attribute measurement in high-speed railway operation. As while as analyzing the operation data of 10 pivotal railway lines in China, this paper respectively uses the rough set-based measurement model and value function model (one model for calculating the safety value) for calculating the operation safety value. The calculation result shows that the curve of safety value with the proposed method has smaller error and greater stability than the value function method’s, which verifies the feasibility and effectiveness.

## Introduction

China’s high-speed railways have been built since 1998, and the first high-speed railway began operating in 2003. The total operating miles in China has increased to 22,000 km now. However, the corresponding development and manufacture technologies are still immature and the relevant operation experience is insufficient in the field of high-speed railway operation [1–3]. For example, the recent Wenzhou train crash accident on July 23, 2011 poses an extreme challenge to the railway safety operation. Therefore it is urgent to make an elaborate inspection of various factors that influence high-speed railway operation so as to ensure safety [4]. Moreover, some relevant measurement results should be conducted and refined to enhance the protection system and improve the operation management level [5].

It is believed that the comprehensive measurement for high-speed railway under the safety operation situation is a principal way to improve management level [6]. Current researches on the safety evaluation of high-speed railway operation could be mainly divided into two categories: one is emergency prediction of macro disaster and another is impact mechanism analysis of micro environmental factors [7–10]. For the first category, some key problems of safety evaluation have been outlined, such as alarm threshold, train control mode and high-speed railway warning system. For the second category, the potential factors that cause operation disaster are mainly the following four aspects: personnel, equipment, management and environment. Besides that, some scholars introduced the analytic hierarchy process (AHP) to establish the risk assessment system of high-speed railway [11–12]. The specific discussions of impact mechanism such as rain, wind and other disaster elements in the high-speed railway have been fully considered in the aspect of environmental factors impact mechanism [13–14]. The existing models and methods include D-S evidence theory, expert evaluation, grey correlative grade method and fuzzy comprehensive evaluation method, etc. were well used for evaluating the safety degree [15–18]. Whereas the result of D-S evidence theory usually appears some counterintuitive conclusions when dealing with the conflict evidences under the normalization process. Expert evaluation has the advantage of fast prediction, but it can not reflect the objective reality necessarily. Grey correlative grade method doesn’t need many examples and specific distribution regularities so that it has the advantage of small computation. However, the optimal value of each factor is difficult to be determined with subjectivity. Fuzzy comprehensive evaluation method also has the problems of strong subjectivity and complicated calculation. Value function is a value between decision-maker’s subjective feeling and gains or losses, which has been applied in the decision-making science. This method is very effective in dealing with the attribute membership problem, and it is also well used for evaluating the safety of traffic network [19–20].

Based on the above analysis, this paper sets up a measurement model for evaluating operation safety based on the assumption of measurement factors and results, which has combined the rough theory and the measurement matrix and reduction method in the uncertainty theory. The rough set theory has been proved its effectiveness on machine learning, intelligent systems, inductive reasoning, decision analysis, and expert systems [21]. In especial it provides better performance in selecting the clustering attribute in terms of purity, entropy, accuracy and others [22]. In order to increase the objectivity of decision-making process, this paper adopts the fuzzy-distance method [23] to determine the weight coefficient of measurement index of high-speed railway safety operation.

## Measurement index system of safety operation

According to the description of the rough set theory, the knowledge inference for railway operation is a kind of approaches that calculates all minimal decision methods, which is based on the given condition characteristics and result characteristics of knowledge system [24]. As the principle of measurement system described, the system can measure the changes in different situations during different time periods and measure the differences between each operation situation at the same time [25]. Thus the measurement model is established for evaluating the safety situation due to the measurement index system, which is an important foundation for describing and measuring the high-speed railway safety operation. Some relative measurement indexes are mainly simplified by the rough set theory in this chapter, and the main factors that influence the evaluation of safety operation are picked out.

### 2.1 Measurement index system

The concept of safety operation is dynamic, and it is constantly changing with the development of society [26]. The measurement index system about high-speed railway safety operation could be intensively built in accordance with the principle of comprehensiveness, practicability, measurability and comparability. Through comprehensive analysis of the operation characteristics of high-speed railway, the relative factors which influence the operation safety could be divided into 3 major types:

1. Human factor. Staff’s comprehensive quality, level of education and attitude for work are the key factors to ensure safety operation. It is reported that the ratio of human errors causing accidents is over 70%. Therefore, the human factor is the key element of railway safety operation.
2. Equipment factor. Suitable equipment is not only the material basis but also the important guarantee for safety operation of high-speed railway.
3. Environment factor. The environment includes not only social, natural and working condition and environment but also the surrounding space and natural human environment, which composes of all the production facilities. In order to decrease every harmful factor as far as possible in the environment factor, the potential unsafe factor should be found out and the level scores should be defined by the expert experience.

The measurement index system of high-speed railway safety operation and its level scores are elaborated in Table 1.

**Table 1:** The measurement index system of high-speed railway safety operation.

The measurement index			Safety level				
			Very safe	Safe	General	Unsafe	Very unsafe
**Staff index**	*I*_1_	Technical ability	5	4	3	2	1
	*I*_2_	Professional skill	5	4	3	2	1
	*I*_3_	Work attitude	5	4	3	2	1
	*I*_4_	Safety awareness	5	4	3	2	1
**Vehicle index**	*I*_5_	Transmission technology	5	4	3	2	1
	*I*_6_	Braking technology	5	4	3	2	1
	*I*_7_	Bodywork technology	5	4	3	2	1
	*I*_8_	Safety technology	5	4	3	2	1
**Equipment Index**	*I*_9_	Power supply system	5	4	3	2	1
	*I*_10_	Operation control system	5	4	3	2	1
	*I*_11_	Safety equipment system	5	4	3	2	1
	*I*_12_	Operation monitoring system	5	4	3	2	1
**Environment Index**	*I*_13_	Wind speed control system	5	4	3	2	1
	*I*_14_	Rainfall monitoring system	5	4	3	2	1
	*I*_15_	Debris flow monitoring system	5	4	3	2	1
	*I*_16_	Earthquake monitoring system	5	4	3	2	1

### 2.2 Level definition of safety operation evaluation

The classification standard of measurement index respectively takes the actual index of national standard or the average level of abroad and domestic high-speed railway passenger-carrying situations as the medium level, and other levels are acquired by the examination and extrapolation. In this paper, the measurement index could be classified into 5 levels: very safe, safe, general, unsafe and very unsafe so that a relative interval of each level above could be set up, and the specific situation is displayed in Table 1. For simplifying the formula computing of the rough set theory, the original digits are introduced to the discretization process. Putting 16 measurement indexes as the condition attribution, and the condition attribution is classified into 5 levels like 5, 4, 3, 2, 1, which represent very safe, safe, general, unsafe and very unsafe respectively. Then classifying the comprehensive measurement results into 5 groups as the decision attribution, which are noted as *D* = {5, 4, 3, 2, 1}, and they represent very safe, safe, general, unsafe and very unsafe respectively.

## Measurement model of safety operation

It is well known that the rough set theory was provided as a way to study the incomplete and unsure knowledge, and the method can simplify the calculations [21–22]. A comprehensive measurement method is proposed according to the rough set theory which based on some relative national standards and statistical data. Firstly defining *x*_1_, *x*_2_, ⋯, *x*_*n*_ as *n* high-speed railway lines, which are described as *X* = {*x*_1_, *x*_2_, ⋯, *x*_*n*_}. Then defining *I* = {*I*_1_, *I*_2_, ⋯, *I*_16_}as the index set in the measurement index, where *x*_*ij*_ is the measurement value of high-speed railway line *x*_*i*_ of the measurement index *I*_*j*_. The measurement space is mainly described the size of subset which is composed by a number of elements. Thus defining *C* = {*c*_1_, *c*_2_, ⋯, *c*_*k*_} as the measure space, where *c*_*k*_(1 ≤ *k* ≤ *K*) means the *k*-th measurement degree of safety operation. At last, the calculated measurement results combine qualitative information, history accident data with the present information.

### 3.1 Measurement degree of the single index

The measurement value *x*_*ij*_, which means the high-speed railway line *x*_*i*_ of measurement index *I*_*j*_, all of *x*_*ij*_ are not the same. Defining the degree *μ*_*ijk*_ = *μ*(*x*_*ij*_ ∈ *c*_*k*_) as the measurement level of *c*_*k*_, which is the *k*-th level of high-speed railway line *x*_*i*_, and *μ*_*ijk*_ must satisfy the condition: 0 ≤ *μ*_*ijk*_ ≤ 1, $\mu (x_{ij}\in \overset{K}{\underset{k=1}{\cup }}c_{k})=\sum_{k=1}^{K}\mu (x_{ij}\in c_{k})$, *μ*(*x*_*ij*_ ∈ *c*) = 1, where *i* = 1, 2, ⋯, *n*, *j* = 1, 2, ⋯, 16 and *k* = 1, 2, ⋯, *K*. Since the railway line *x*_*i*_ are calibrated by *j* measurement indexes, and one measurement index is divided into *k* measurement degrees, all of *μ*_*ijk*_ can be constructed as

$${\left(\mu_{ijk}\right)}_{m\times K}=\left[\begin{array}{cccc} \mu_{i11} & \mu_{i12} & \cdots & \mu_{i1K}\\ \mu_{i21} & \mu_{i22} & \cdots & \mu_{i2K}\\ \vdots & \vdots & \cdots & \vdots \\ \mu_{im1} & \mu_{im2} & \cdots & \mu_{imK} \end{array}\right],(i=1,2,\cdots ,n)$$ (1)

(*μ*_*ijk*_)_*m*×*K*_ is the single measurement matrix of high-speed railway line *x*_*i*_, *μ*_*ijk*_ is the unknown measurement matrix, and *μ*_*ij*_(1 ≤ *j* ≤ 16) represents the unknown measurement value of line *x*_*i*_.

### 3.2 Weight coefficient of measurement index

Due to the complexity of high-speed railway operation system, the more training samples could not be gotten so that the objective weighting method could not be used to obtain the weighting coefficient of measurement index [27]. This paper adopts the fuzzy-distance method to determine the weight coefficient of measurement index of high-speed railway safety operation for increasing the objectivity in the decision-making process. Supposing that *w*_*j*_ is the weighting coefficient of measurement index *I*_*j*_ of high-speed railway safety operation, *w*_*j*_ can be described as

$$w_{j}=\frac{1}{m}\sum_{i=1}^{m}\left|x_{ij}-\frac{1}{m}\sum_{i=1}^{m}x_{ij}\right|\cdot {\left[\sum_{j=1}^{n}\left[\frac{1}{m}\sum_{i=1}^{m}\left|x_{ij}-\frac{1}{m}\sum_{i=1}^{m}x_{ij}\right|\right]\right]}^{-1}$$ (2)

Where *x*_*ij*_ is the measurement value of railway line *x*_*i*_ of the measurement index *I*_*j*_.

### 3.3 Comprehensive measurement of safety operation

The single measurement matrix of high-speed railway line *x*_*i*_ could be obtained by formula (1), and the weight coefficient of measurement index could be obtained by formula (2), thus *μ*^*i*^ could be figured out as

$$\begin{array}{ll} \mu^{i} & =W\cdot {\left(\mu_{ijk}\right)}_{m\times K}\\ & =(w_{1},w_{2},\cdots ,w_{m})\cdot \left[\begin{array}{cccc} \mu_{i11} & \mu_{i12} & \cdots & \mu_{i1K}\\ \mu_{i21} & \mu_{i22} & \cdots & \mu_{i2K}\\ \vdots & \vdots & \cdots & \vdots \\ \mu_{im1} & \mu_{im2} & \cdots & \mu_{imK} \end{array}\right]\\ & =(\mu_{i1},\mu_{i2},\cdots ,\mu_{iK}) \end{array}$$ (3)

Where *μ*^*i*^ is the measurement vector of safety operation of high-speed railway line *x*_*i*_.

### 3.4 Measurement principles of safety operation

The measurement level classification of high-speed railway safety operation is arranged in order, and the measurement level *c*_*k*_ is better than the measurement level *c*_*k+*1_. Therefore, it is not suitable that using the criterion of confidence coefficient to evaluate the maximum measurement level. For dealing with the problem above, we make two principles for the safe operation measurement.

#### Principle 1

Defining *k*_*o*_ as the measurement level and defining *λ*(*λ* > 0.5) as the confidence coefficient of high-speed railway safety operation. *k*_*o*_ could be described as

$$k_{o}=\underset{k}{\text{min}}\left[(\sum_{l=1}^{k}\mu_{il})\geq \lambda ,k=1,2,\cdots ,K\right]$$ (4)

Estimating the high-speed railway line *x*_*i*_ whether satisfies the measurement level $c_{k_{o}}$ of *k*_*o*_ through formula (4).

#### Principle 2

The orderly values of high-speed railway safety operation could be described as

$$p_{i}=\text{max}(\mu_{i1},\mu_{i2},\cdots ,\mu_{iK})$$ (5)

The comprehensive measurement value *p*_*i*_ of the high-speed railway line *x*_*i*_ could be acquired by formula (5). According to the description of measurement model, *p*_*i*_ is bigger which means the operation situation of high-speed railway is better. On the contrary, *p*_*i*_ is smaller which means the situation is worse.

## Case analysis

China is extensively developing its infrastructure of high-speed railway and drawing up a plan to cover the major economic areas through the high-speed railway networks with four horizontal and four vertical lines in the next several years. Table 2 gives an overview of the safety situation of 10 high-speed railways which has been analyzed.

**Table 2:** Overview of 10 high-speed railways.

Code	Line Name	Design speed	Line length	The "four vertical and four horizontal" Line
Passenger Line *R*_*1*_	Hening Line	250*km/h*	166*km*	Huhanrong Passenger Line
Passenger Line *R*_*2*_	Jiaji Line	250*km/h*	364*km*	Qingtai Passenger Line
Passenger Line *R*_*3*_	ShiTai Line	250*km/h*	190*km*	Qingtai Passenger Line
Passenger Line *R*_*4*_	Hewu Line	250*km/h*	351*km*	Huhanrong Passenger Line
Passenger Line *R*_*5*_	Wenfu Line	250*km/h*	298*km*	Southeast Coast Passenger Line
Passenger Line *R*_*6*_	Yongtaiwen Line	350*km/h*	268*km*	Southeast Coast Passenger Line
Passenger Line *R*_*7*_	ShangHang Line	350*km/h*	158*km*	Southeast Coast Passenger Line
Passenger Line *R*_*8*_	FuXia Line	200*km/h*	273*km*	Southeast Coast Passenger Line
Passenger Line *R*_*9*_	Yiwan Line	200*km/h*	377*km*	Huhanrong Passenger Line
Passenger Line *R*_*10*_	Hanyee Line	200*km/h*	293*km*	Huhanrong Passenger Line

The detail operation data (including the mileage, energy consumption, performance quality of train staffs, etc.) of the 10 railway lines in the “four verticals and four horizontals” are provided by Shanghai Railway Administration (SRA), parts of data can be found in the official site of SRA. Besides that, the operation condition of the 10 lines: *R*_*1*_, *R*_*2*_, *R*_*3*_, *R*_*4*_, *R*_*5*_, *R*_*6*_, *R*_*7*_, *R*_*8*_, *R*_*9*_, *R*_*10*_ have investigated by our research team in August 2013.

Dividing the investigated original data about the 10 railway lines into 16 measurement indexes as the criterion of Table 1 and then organizing 4 experts of different research areas in environment engineering, transport engineering, social engineering and mechanical engineering to comprehensively judge the safety scores with their subjective experience. The key of judging criterion is to analyze the number of accidents, energy consumption, academic diplomas and skill levels of operators, economical levels of cities, etc. Finally, the decision-making table is shown in Table 3.

**Table 3:** The decision-making table of high-speed railway.

Measurement index		*R*_*1*_	*R*_*2*_	*R*_*3*_	*R*_*4*_	*R*_*5*_	*R*_*6*_	*R*_*7*_	*R*_*8*_	*R*_*9*_	*R*_*10*_
**Investigated value of the measurement index of high- speed railway safety operation**	*I*_*1*_	2	3	2	3	2	2	3	3	3	2
	*I*_*2*_	3	4	3	3	2	2	3	2	2	4
	*I*_*3*_	3	3	3	4	4	2	3	3	2	4
	*I*_*4*_	2	3	3	4	3	4	2	2	4	4
	*I*_*5*_	3	3	3	4	3	2	2	3	3	3
	*I*_*6*_	4	4	3	3	3	3	2	4	4	3
	*I*_*7*_	2	2	3	3	3	3	4	2	2	2
	*I*_*8*_	3	3	3	4	3	3	4	4	3	3
	*I*_*9*_	2	2	2	3	3	3	4	3	3	3
	*I*_*10*_	2	3	3	3	3	2	3	3	4	4
	*I*_*11*_	3	2	4	3	3	2	4	3	3	3
	*I*_*12*_	3	3	3	3	3	4	2	3	3	3
	*I*_*13*_	2	3	3	3	4	2	4	3	3	2
	*I*_*14*_	3	3	3	3	4	4	2	2	2	4
	*I*_*15*_	3	3	3	4	3	3	4	4	4	3
	*I*_*16*_	3	4	3	4	2	3	4	2	2	3
**Expert assessment scores**	1	84	87	76	80	81	80	78	73	87	81
	2	83	85	78	79	87	88	76	78	81	82
	3	79	83	80	78	84	84	71	88	84	80
	Average	82	85	78	79	84	84	75	79	84	81
	*D*	4	4	3	3	4	4	3	3	4	4

### 4.1 Measurement of high-speed railway safety operation based on the rough set

The redundant and core attribution of safety operation are obtained from Table 3. Due to the complexity of measurement index system and calculation, it is necessary to simplify the attribution factors for increasing the calculation accuracy and simplifying the calculation process. After comprehensively evaluating the important degree of the 16 measurement indexes, as while as using the reduction method of the rough set theory for reducing the contents of measurement index system to 6 factors such as the safety education level, total work attitude, motor driving technology, safety facilities system, rainfall monitoring system and earthquake monitoring system. The relative measurement index system is obtained as Table 4 displayed and the specific information in Table 4 has removed some redundant messages so as to obtain more operationally reliable results.

**Table 4:** The measurement indexes of safety operations after reduction.

Target layer	Measurement index of high-speed railway safety operation	
**The measurement index of high-speed railway safety operation**	*I*_*1*_	Technical ability
	*I*_*4*_	Safety awareness
	*I*_*6*_	Braking technology
	*I*_*8*_	Safety technology
	*I*_*9*_	Power supply system
	*I*_*10*_	Operation control system
	*I*_*12*_	Operation monitoring system
	*I*_*13*_	Wind speed control system
	*I*_*15*_	Debris flow monitoring system

According to the measurement model for safety operation, the evaluation values of the 10 high-speed railways lines *R*_*1*_, *R*_*2*_, *R*_*3*_, *R*_*4*_, *R*_*5*_, *R*_*6*_, *R*_*7*_, *R*_*8*_, *R*_*9*,_ *R*_*10*_ are given, and there are 9 measurement factors and each evaluated object will get 5 points. According to the data of the evaluated object such as line *R*_*1*_, the single index of measurement matrix *μ*_1*jk*_ of high-speed railway safety operation is acquired as

$$\mu_{1jk}=\left[\begin{array}{ccccc} 0.3251 & 0.2745 & 0.2113 & 0.1406 & 0.0481\\ 0.1413 & 0.2322 & 0.3111 & 0.2332 & 0.0822\\ 0.1035 & 0.2439 & 0.3591 & 0.2319 & 0.0616\\ 0.1258 & 0.1356 & 0.3332 & 0.3227 & 0.0827\\ 0.1501 & 0.2311 & 0.3131 & 0.1266 & 0.1781\\ 0.1336 & 0.2122 & 0.2693 & 0.2357 & 0.1492\\ 0.1513 & 0.3341 & 0.3203 & 0.1026 & 0.1017\\ 0.0762 & 0.1683 & 0.3571 & 0.2339 & 0.1645\\ 0.0817 & 0.1175 & 0.4155 & 0.3236 & 0.0617 \end{array}\right]$$ (6)

The weight vector of measurement index for high-speed railway safety operation could be got through formula (2), so there is

W=(0.1117,0.1103,0.1111,0.1115,0.1012,0.1213,0.1203,0.1104,0.1012) (7)

Then the measurement vector of high-speed railway *R*_*1*_ is obtained by formula (3).

$$\mu^{1}=W\cdot \mu_{1jk}=(0.1437,0.2183,0.3293,0.2155,0.1033)$$ (8)

Obtaining *λ* = 0.6 from formula (4) when *k*_*o*_ = 3, it has a judge process

0.3293+0.2183+0.1033=0.6509>0.6 (9)

Therefore, the safety operation situation of *R*_*1*_ belongs to the third level. Through formula (5), the measurement value of line *R*_*1*_ is *p*_1_ = 0.3293. Similarly, the other values of safety operation about lines: *R*_*2*_, *R*_*3*_, *R*_*4*_, *R*_*5*_, *R*_*6*_, *R*_*7*_, *R*_*8*_, *R*_*9*,_ *R*_*10*_ could be calculated and they are showed in Table 5.

**Table 5:** The measurement vectors of safety operation.

Name	Evaluation vector of safety operation of high-speed railway	Sort values	Safety level
Passenger Line *R*_*1*_	*μ*^1^ = (0.1437, 0.2183, 0.3293, 0.2155, 0.1033)	*p*_1_ = 0.3293	3
Passenger Line *R*_*2*_	*μ*^2^ = (0.1563, 0.1793, 0.2984, 0.2173, 0.1113)	*p*_2_ = 0.2984	3
Passenger Line *R*_*3*_	*μ*^3^ = (0.1247, 0.2078, 0.3083, 0.1667, 0.0987)	*p*_3_ = 0.3083	3
Passenger Line *R*_*4*_	*μ*^4^ = (0.1421, 0.2278, 0.3187, 0.2017, 0.1782)	*p*_4_ = 0.3187	3
Passenger Line *R*_*5*_	*μ*^5^ = (0.1227, 0.2323, 0.3376, 0.2152, 0.1013)	*p*_5_ = 0.3376	3
Passenger Line *R*_*6*_	*μ*^6^ = (0.1551, 0.2313, 0.3501, 0.1203, 0.1114)	*p*_6_ = 0.3501	3
Passenger Line *R*_*7*_	*μ*^7^ = (0.1278, 0.2059, 0.3392, 0.1807, 0.1623)	*p*_7_ = 0.3392	3
Passenger Line *R*_*8*_	*μ*^8^ = (0.1217, 0.2237, 0.3224, 0.2001, 0.1611)	*p*_8_ = 0.3224	3
Passenger Line *R*_*9*_	*μ*^9^ = (0.1199, 0.2339, 0.3021, 0.1999, 0.1315)	*p*_9_ = 0.3021	3
Passenger Line *R*_*10*_	*μ*^10^ = (0.1617, 0.2071, 0.3207, 0.2114, 0.1051)	*p*_10_ = 0.3207	3

**Fig 1:**
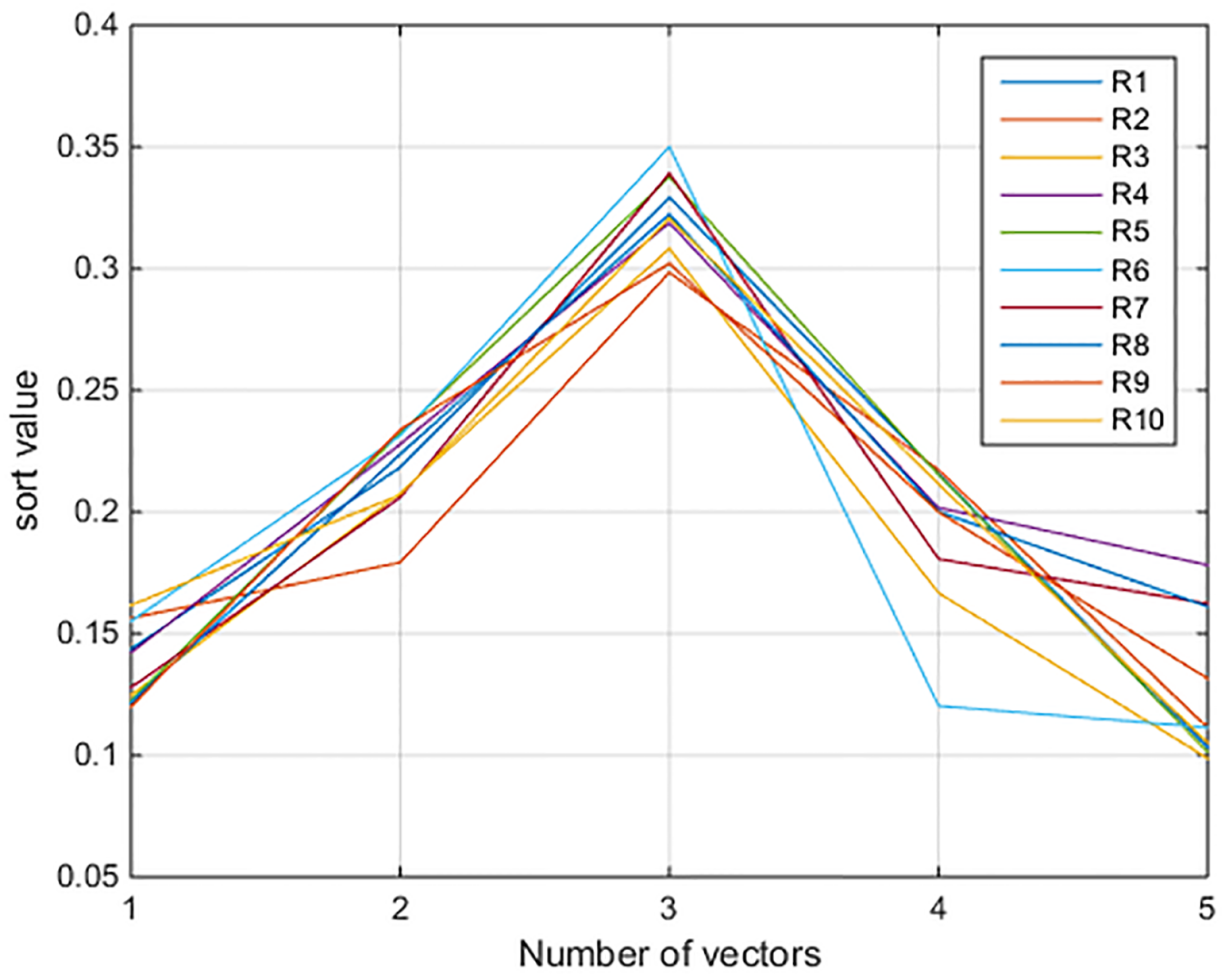
The evaluation values of high-speed railways.

**Fig 2:**
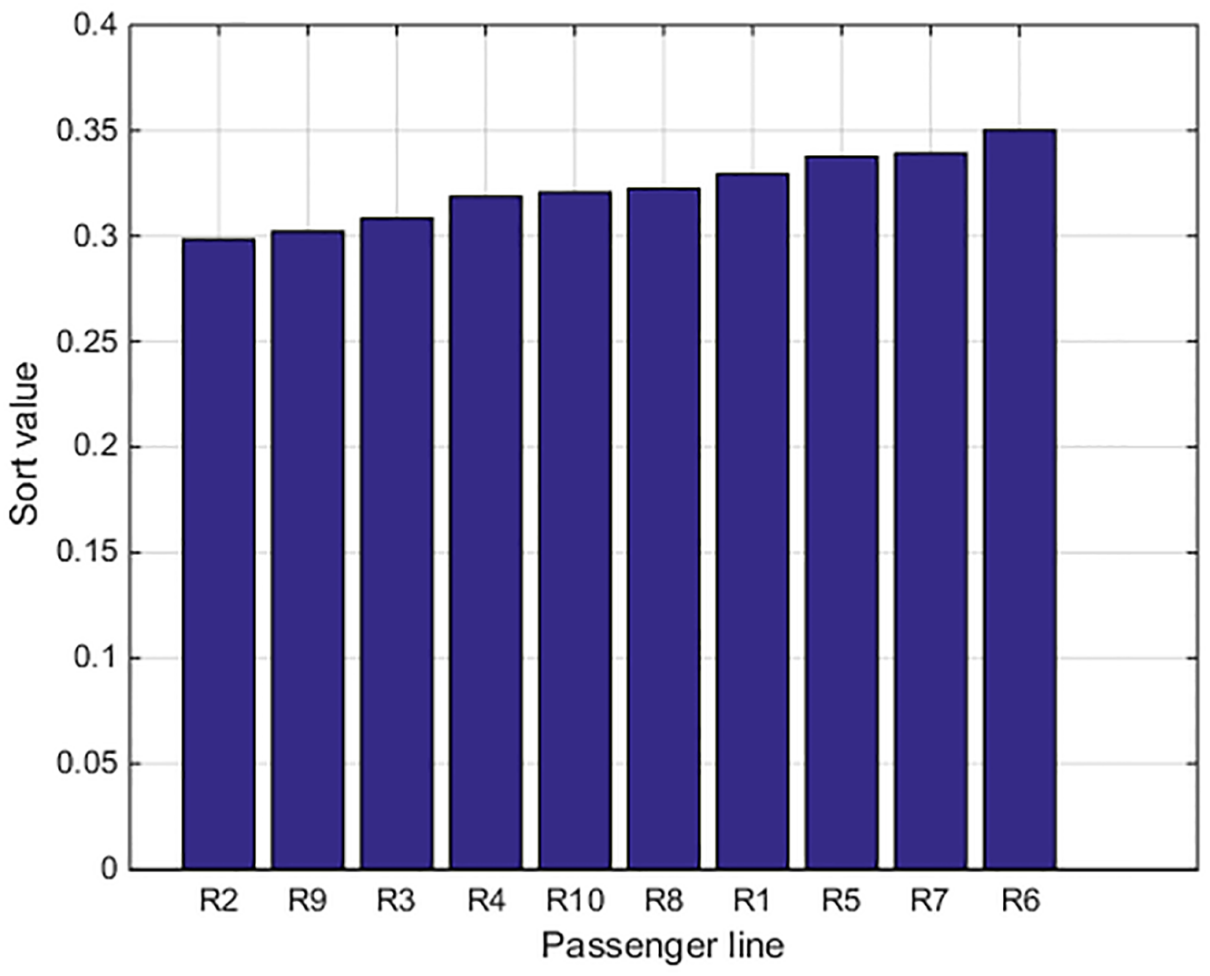
The order of the sort values about passenger lines.

The ordination graphs of them are shown in Figs 1 and 2.

As one can see from Fig 1, the rough set-based measurement model calculates the sort values in different vectors of evaluation as while as the maximum values of the 10 lines are concentrated on the third vector, in other words, the safety operation situation of the 10 lines are all belonging to the third level (General level). Fig 2 shows the sort values, obviously the order of operation safety of the 10 high-speed railway lines is *R*_*6*_> *R*_*7*_> *R*_*5*_> *R*_*1*_> *R*_*8*_> *R*_*10*_> *R*_*4*_> *R*_*3*_> *R*_*9*_> *R*_*2*_.

### 4.2 Measurement of high-speed railway safety operation by value function

In order to measure the operation safety, it is very practical for using value function to analyze and evaluate high-speed railway. Value function is a flexible and practical method, its main characteristic is that it could combine qualitative with quantitative in decision-making process. Assuming that $f_{I_{j}}(x_{ij})$ is the value function of measurement index *I*_*j*_. Here *w*_*j*_ is the weight coefficient of measurement index *I*_*j*_, and $\sum_{j=1}^{16}w_{j}=1$. Then the calculation procedure is presented as follow:

#### Step 1

The weight coefficient of measurement indicators can be determined by formula (2).

$$\begin{array}{l} w_{1}=0.0625,w_{2}=0.0603,w_{3}=0.0647,w_{4}=0.0651,w_{5}=0.0599,w_{6}=0.0632,\\ w_{7}=0.0618,w_{8}=0.0597,w_{9}=0.0653,w_{10}=0.0641,w_{11}=0.0609,w_{12}=0.0625,\\ w_{13}=0.0611,w_{14}=0.0639,w_{15}=0.0592,w_{16}=0.0658 \end{array}$$ (10)

#### Step 2

The value of high-speed railway safety operation can be obtained by formula (11)

fIj(xij)={x*(x¯x*)2k1⋅exp{xij(maxjxij)−1⋅ln(x¯x*)−2k1},Ij∈J+x*(x¯x*)2k2⋅exp{(maxjxij−xij)⋅(maxjxij)−1⋅ln(x¯x*)−2k1},Ij∈J− (11)

Where $k_{1}=\underset{j}{\text{max}}x_{ij}/\left(\underset{j}{\text{max}}x_{ij}-\underset{j}{\text{min}}x_{ij}\right)$, $k_{2}=r_{j}/\left(\underset{j}{\text{max}}x_{ij}-\underset{j}{\text{min}}x_{ij}\right)$, x* is the value of the optimal indicator, $\bar{x}$ is the average value of the indicators. What’s more, *J*^+^ is the benefit-type indicators set, and *J*^−^ is the cost-type indicators set.

The values of safety operation of the 10 high speed railway lines are given in Table 6, and all the investigated data are consistent with the case analysis of rough set model.

**Table 6:** The value of safety operation of the 10 high speed railway lines.

Index	Line *R*_*1*_	Line *R*_*2*_	Line *R*_*3*_	Line *R*_*4*_	Line *R*_*5*_	Line *R*_*6*_	Line *R*_*7*_	Line *R*_*8*_	Line *R*_*9*_	Line *R*_*10*_
*I*_*1*_	0.9372	0.7381	0.4372	0.3227	0.6321	0.8113	0.5231	0.7239	0.8224	0.6178
*I*_*2*_	0.8713	0.7913	0.4068	0.3019	0.6074	0.7994	0.5217	0.7044	0.8117	0.6104
*I*_*3*_	0.9014	0.6992	0.4183	0.3774	0.5998	0.8019	0.5033	0.7213	0.8103	0.6139
*I*_*4*_	0.7991	0.7134	0.4627	0.3118	0.6027	0.8104	0.5129	0.7127	0.8127	0.6023
*I*_*5*_	0.8925	0.6881	0.5012	0.3009	0.6108	0.7998	0.5083	0.7093	0.8174	0.6005
*I*_*6*_	0.9013	0.6935	0.3997	0.2994	0.6217	0.8027	0.5209	0.6997	0.8217	0.5993
*I*_*7*_	0.9372	0.7036	0.4331	0.2909	0.6233	0.8093	0.4997	0.7017	0.8203	0.5927
*I*_*8*_	0.7927	0.7454	0.5001	0.3071	0.5991	0.8102	0.4988	0.7082	0.7991	0.6012
*I*_*9*_	0.7933	0.7822	0.4723	0.3109	0.6003	0.7993	0.5003	0.7124	0.7904	0.6127
*I*_*10*_	0.8451	0.7717	0.4614	0.3098	0.6015	0.8005	0.5019	0.7225	0.8003	0.6023
*I*_*11*_	0.9092	0.6936	0.4113	0.3204	0.6084	0.8013	0.5116	0.6909	0.8079	0.6009
*I*_*12*_	0.7968	0.6982	0.5003	0.2995	0.6127	0.8014	0.5223	0.7083	0.8102	0.6011
*I*_*13*_	0.8931	0.7034	0.4789	0.3004	0.6194	0.7992	0.5234	0.7113	0.8134	0.6077
*I*_*14*_	0.7889	0.7315	0.4414	0.3187	0.6208	0.7989	0.4987	0.7188	0.8142	0.5991
*I*_*15*_	0.8617	0.7133	0.4237	0.3204	0.6271	0.8101	0.4994	0.7204	0.8153	0.6023
*I*_*16*_	0.8972	0.7271	0.4891	0.3003	0.6371	0.8094	0.5023	0.7213	0.7994	0.6109

#### Step3

The comprehensive measurement value of high-speed railways safety operation is determined by the equation $U(R_{i})=\sum_{j=1}^{16}w_{j}f_{I_{j}}(x_{ij})$. So

$$\begin{array}{l} U(R_{1})=0.863294,U(R_{2})=0.724769,U(R_{3})=0.452411,U(R_{4})=0.312185,\\ U(R_{5})=0.614001,U(R_{6})=0.804063,U(R_{7})=0.509237,U(R_{8})=0.711814,\\ U(R_{9})=0.810317,U(R_{10})=0.604762 \end{array}$$ (12)

According to *U* (*R*_*i*_) of the comprehensive measurement, the safety ranking of high-speed railway line is *R*_*1*_ > *R*_*9*_> *R*_*6*_> *R*_*2*_> *R*_*8*_> *R*_*5*_> *R*_*10*_> *R*_*7*_ > *R*_*3*_ >*R*_*4*_.

### 4.3 Comparative analysis about the calculation results of two measurement models

**Fig 3:**
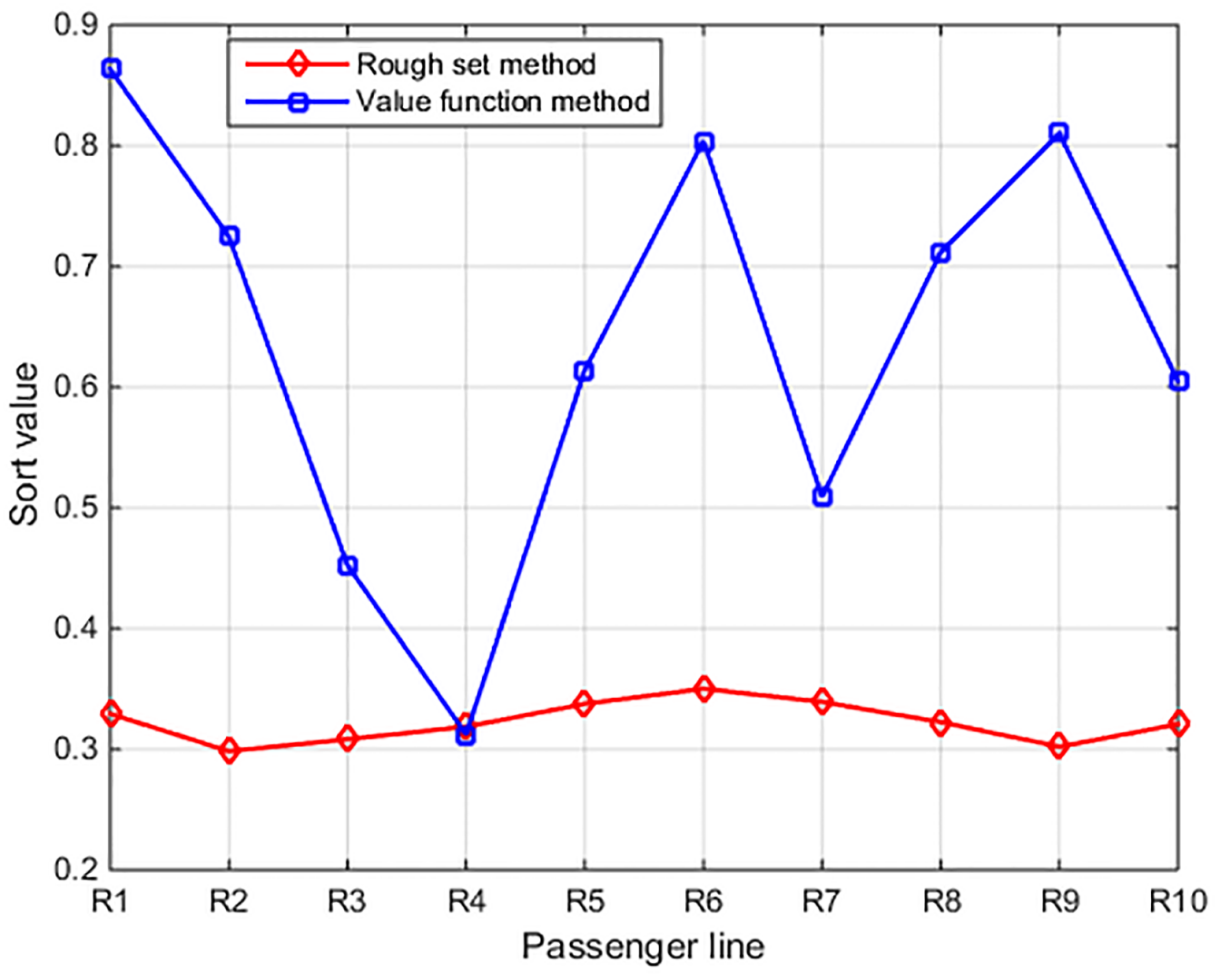
The order of sort values about the 10 high-speed railway lines by two measurement models.

Based on the case analysis in the last two chapters, the calculation results of two measurement models are different. The priority relationship about the safety ranking of the 10 high-speed railway lines is *R*_*6*_> *R*_*7*_> *R*_*5*_> *R*_*1*_> *R*_*8*_> *R*_*10*_> *R*_*4*_> *R*_*3*_> *R*_*9*_> *R*_*2*_, the calculation result of two models are shown in Fig 3.

The chart of Fig 3 by MATLAB shows the 10 high-speed railways operation safety between the two measurement models. The calculation error is an efficacious value to present the value changes of the evaluation result. The calculation error between the maximum and minimum values of rough set-based model is 0.0517, and the calculation error of value function-based model is 0.5511. Furthermore, the relative error of proposed method is 0.1477, and the relative error of value function-based model is 0.6384. It is obvious that the curve of rough set-based measurement model is more smooth and steady than the value function one. Thus the proposed rough set-based measurement model has more evaluation accuracy than the value function-based model.

## Conclusions

Safety degree is an evaluation criterion that reflects the development state and comprehensive safety level of high-speed railway. It also can provide the parameters that guide the decision-maker to manage and change the condition of railway. Based on the rough set theory, a deep insight of the effective model is provided for evaluating the safety of high-speed railway. The paper sets up a new measurement index system of high-speed railways safety operation in scientific way and simplifies the measurement factors that influence the safety of high-speed railway. The proposed model overcomes the defects of single measurement index, and obtains the evaluation result in accordance with the single index measurement matrix. In addition, the proposed method has smaller error and greater stability than the value function method one. Thus, the measurement model is of great theoretical and practical significance for high-speed railway safety operation, and it has a great application prospect in the field of safety evaluation.
